# New hopes for the breast cancer treatment: perspectives on the oncolytic virus therapy

**DOI:** 10.3389/fimmu.2024.1375433

**Published:** 2024-03-21

**Authors:** Hanna Chowaniec, Antonina Ślubowska, Magdalena Mroczek, Martyna Borowczyk, Małgorzata Braszka, Grzegorz Dworacki, Paula Dobosz, Mateusz Wichtowski

**Affiliations:** ^1^ Department of Immunology, Poznan University of Medical Sciences, Poznan, Poland; ^2^ Department of Biostatistics and Research Methodology, Faculty of Medicine, Collegium Medicum, Cardinal Stefan Wyszynski University of Warsaw, Warsaw, Poland; ^3^ Department of Neurology, University Hospital Basel, Univeristy of Basel, Basel, Switzerland; ^4^ Department of Endocrinology, Metabolism and Internal Medicine, Poznan University of Medical Sciences, Poznan, Poland; ^5^ Faculty of Medical Sciences, University College London Medical School, London, United Kingdom; ^6^ Chair of Patomorphology and Clinical Immunology, Poznań University of Medical Sciences, Poznan, Poland; ^7^ University Centre of Cancer Diagnostics, Poznan University of Medical Sciences, Poznan, Poland; ^8^ Institute of Genetics and Biotechnology, Faculty of Biology, University of Warsaw, Warsaw, Poland; ^9^ Surgical Oncology Clinic, Institute of Oncology, Poznan University of Medical Sciences, Poznan, Poland

**Keywords:** breast cancer, immunotherapy, oncolytic virus therapy, tumour microenvironment, TME

## Abstract

Oncolytic virus (OV) therapy has emerged as a promising frontier in cancer treatment, especially for solid tumours. While immunotherapies like immune checkpoint inhibitors and CAR-T cells have demonstrated impressive results, their limitations in inducing complete tumour regression have spurred researchers to explore new approaches targeting tumours resistant to current immunotherapies. OVs, both natural and genetically engineered, selectively replicate within cancer cells, inducing their lysis while sparing normal tissues. Recent advancements in clinical research and genetic engineering have enabled the development of targeted viruses that modify the tumour microenvironment, triggering anti-tumour immune responses and exhibiting synergistic effects with other cancer therapies. Several OVs have been studied for breast cancer treatment, including adenovirus, protoparvovirus, vaccinia virus, reovirus, and herpes simplex virus type I (HSV-1). These viruses have been modified or engineered to enhance their tumour-selective replication, reduce toxicity, and improve oncolytic properties.Newer generations of OVs, such as Oncoviron and Delta-24-RGD adenovirus, exhibit heightened replication selectivity and enhanced anticancer effects, particularly in breast cancer models. Clinical trials have explored the efficacy and safety of various OVs in treating different cancers, including melanoma, nasopharyngeal carcinoma, head and neck cancer, and gynecologic malignancies. Notably, Talimogene laherparepvec (T-VEC) and Oncorine have. been approved for advanced melanoma and nasopharyngeal carcinoma, respectively. However, adverse effects have been reported in some cases, including flu-like symptoms and rare instances of severe complications such as fistula formation. Although no OV has been approved specifically for breast cancer treatment, ongoing preclinical clinical trials focus on four groups of viruses. While mild adverse effects like low-grade fever and nausea have been observed, the effectiveness of OV monotherapy in breast cancer remains insufficient. Combination strategies integrating OVs with chemotherapy, radiotherapy, or immunotherapy, show promise in improving therapeutic outcomes. Oncolytic virus therapy holds substantial potential in breast cancer treatment, demonstrating safety in trials. Multi-approach strategies combining OVs with conventional therapies exhibit more promising therapeutic effects than monotherapy, signalling a hopeful future for OV-based breast cancer treatments.

## 1 Introduction

Oncolytic viruses (OVs) are the main subject of interest in multiple ongoing clinical trials in many cancer types. Effects in the field of immunotherapy for solid tumours are promising and mainly focussed on the field of immune checkpoint inhibitors and CAR-T cells (1). Despite these therapies having the potential to achieve full tumour regression, there is a number of patients whose response to the treatment is limited (2), fostering the development of new solutions targeting tumours resistant to the currently available forms of immunotherapy (3). Although the first OV gained the approval of the US Food and Drug Administration (FDA) only in 2015^1^, researchers have been working on developing this form of therapy for decades (4). OV therapy is currently known as ,,a major breakthrough in cancer treatment” (4). What makes OVs so clinically useful is their ability to affect the cancer cells through several different mechanisms (3) as well as their selective, destructible impact only on cancer cells, without destroying physiological tissues in the human body (5, 6). For the past twenty years, many clinical trials have been carried out, with the most used OVs being adenovirus, HSV-1, reovirus, vaccinia virus, and Newcastle disease virus, and only a few of them having been approved for commercial use (7).

## 2 Mechanism of action

OVs may be natural or artificially engineered viruses. They can replicate in cancer cells, leading to the lysis (8). This phenomenon has been observed for years as a spontaneous tumour regression after viral infection in some patients. Recent advances in clinical research and genetic engineering enabled the development of specifically targeted viruses, performing various types of anticancer activity (8). Unlike chemotherapy and radiotherapy, OVs kill cancer cells without harm to normal tissues, which makes them a promising alternative to traditional methods of treatment. OVs have also played a significant role in cancer immunotherapy. They can modify the tumour microenvironment (TME) by triggering an antitumour immune response and having synergistic effects with other anticancer therapies (8).

Oncolytic viruses are divided into two groups, natural weak (wild-type OVs) and genetically modified virus strains. As some mutations typical for cancers, such as in *P53, RB1, PTEN, DCC, RAS*, *P16*, and *VHL* genes, impair the antiviral abilities of cells; they are often suitable targets of OV attacks. Some natural virus strains prefer tumour cells. However, their anticancer effectiveness is limited, and the pathogenicity might be challenging to control. Both the safety and performance of viruses can be increased by genetic manipulations, including gene element regulation, and inserting exogenous genes in engineered recombinant OVs. The examples of possible modifications augmenting the anti-tumour efficacy of OVs are presented in **Table 1**. All of them have been already considered as a potential therapy for breast cancer or its metastases.

**Table 1:** Genes expressed by the genetically engineered OVs that improve their anti-cancer efficacy.

Group	Representatives	Effect	OV examples	References
**Cytokine genes**	*GM-CSF, IFN, interleukin (IL-2, IL-7, IL-12, IL-15, IL-18*, *IL-23, IL-24*) genes	Promotion of the presentation and recognition of TAAs, activation of APCs, increase in CD4+ and CD8+ T cells, boost of the anti-tumour immune response, inhibition of tumour proliferation, metastasis, and angiogenesis	T-vec (HSV-1) OH2 (HSV-2), VG161 (HSV-1), T3011 (HSV-1), TILT-123 (AdV), Cont-VV (VV), OncoViron (AdV), Pexa-vec (VV), CG0070 (AdV), M032 (HSV-1)	(9–14)
**Chemokine genes**	*CCL5, CCL20, CCL21, CXCL4L1, CXCL10* genes			(15)
**Immune costimulatory/coinhibitory molecule genes**	*OX40L, CD30, CD40, 4–1BB genes*	Promotion of the activation and proliferation of tumour-specific T cells	LOAd703 (AdV), Delta-24-RGDOX (AdV)	(16, 17)
**Suicide genes**	*HSV-TK, CD, FCU1 genes*	Transformation of some nontoxic drug precursors into cytotoxic substances (e.g. conversion of nontoxic 5-FC to toxic 5-FU and 5-FUMP by FCU1)	T601 (VV)	(16, 18)
**Tumour suppressors genes**	*P53, PTEN, P16, RB genes*	Enhancement of the inhibitory effect of OVs on tumour cells	HSV-P10	(19)
**Pro-apoptotic genes**	*Apoptin, Lactaptin, TRAIL, SMAC genes*	Apoptosis of tumour cells	p55-hTERT-HRE-TRAIL (AdV)	(20)
**TAA genes**	*CEA, PSA, claudin-6 genes*	Induction of systemic anti-tumour response (OVs act as vaccines)	MVvac2-CLDN6 (MV)	(21)
**Anti-angiogenic genes**	*endostatin, angiostatin, can stain, VEGF receptor 1-Ig fusion protein, VEGF single-chain antibody, VEGF promoter targeted transcriptional inhibitor genes*	Inhibition of tumour angiogenesis and growth	T-TSP-1 (HSV-1), HSV-Endo (HSV-1), VV encoding anti-VEGF single-chain antibody GLAF-1	(22–24)
**Anti-tumour antibody genes**	*anti-PD-1, Bi-specific antibody genes*	Enhancement of overall anti-tumour efficacy, maximization of local concentration of T cells at the tumour site	NG34SCFVPD-1 (HSV-1), NG-641, ICOVIR-15K-cBiTE	(25–27)
**ECM-degrading enzyme genes**	MMP-9, PH20 genes	Degradation of ECM components, increase of intratumoral spread of OVs	GLV-1h255 (VV), VCN-01 (AdV)	(28, 29)

## 3 Types of viruses used in oncolytic trials

Each oncolytic virus has its characteristics that imply the safety profile, influence the possible therapeutic use, and suggest which areas require modifications in a particular case (8). In this review, we decided to focus on selected oncolytic virus strains that are used in breast cancer trials in particular. Their mechanisms of tumour targeting and antitumour activity are presented in **Table 2**.

**Table 2:** Mechanism of action of selected OVs.

Virus	Genetic material	Mechanisms of tumour targeting	Mechanisms of antitumour activity	Human pathogenicity
Adenovirus	dsDNA; does not incorporate into infected cells’ chromosomes	Deletion of the gene essential for efficient viral replication in normal cells (e.g. *E1B-55K*); making replication dependent on the enzyme highly active only in cancer cells (e.g. human telomerase reverse transcriptase); modification of viral capsid protein (e.g. adding RGD peptide to a fibre capsid protein, deletion in the E1A region, creating chimeric hexon capsid protein with adenovirus serotype rare in nature)	Anticancer activity of viral structural protein; adding anticancer immunomodulatory genes (IL-12, IFN-γ and CCL5 genes)	+
Protoparvovirus	ssRNA	Natural tropism to human cancer cells (does not induce cell lysis in non-transformed cells); exploiting receptors overexpressed on cancer cells (eg. transferrin), hijacking aberrant signalling pathways (e.g. Ras pathway)	May lead to apoptotic, non-apoptotic, and lysosome-dependent cell death	–
Vaccinia virus	Large dsDNA, can be inserted with big fragments of transgenes; does not integrate into host cell’s chromosomes	Knocking out the thymidine kinase	Modifying virus to express factors that activate the systemic immune response and inhibit tumour cells (eg. GM-CSF); combining features of two distinct infectious forms to help the virus evading neutralising antibodies	+ (typically very mild infection)
Reovirus	dsRNA	Replication promoted in cells with an activated RAS pathway	Increases PD-L1 expression on tumour cells; stimulates the recruitment of NK cells and reovirus-specific CD8+ T cells to the tumour site; Modifying virus to antagonize inhibitory mechanisms within the TME (e.g. mutations in viral cell attachment protein *σ1* gene)	+ (only some genera)
Herpes simplex virus type 1	dsDNA	Modifying or deleting the genes that are crucial for viral replication in normal cells (e.g. thymidine kinase, ICP34.5, ICP6, ICP47 genes)	Knocking out the ICP47 gene to help activate host antitumour immune response,	+

"+" means YES; "-" means NO.

### 3.1 Adenovirus

Adenovirus is a DNA double-stranded virus that can enter the cells in a receptor-mediated way or through endolysis. Its genomic DNA is released and transferred to the nucleus, where it is replicated but not incorporated into chromosomes. The main efforts in the optimisation design of oncolytic adenoviruses concern restricting their replication selectively to cancer cells together with reducing viral toxicity and enhancing their oncolytic properties.

Selective replication was achieved in ONYX-050 and H101, the prototypes for oncolytic adenoviral therapy, by deletion of the viral *E1B-55K* gene, which is essential for efficient viral replication in normal cells but not in tumour cells, where this process is regulated differently (30). Another approach is to make the adenovirus replication dependent on the enzymes highly active only in cancer cells (31). A good example is tumour-specific replication-competent adenovirus OBP-301, in which the human telomerase reverse transcriptase (the catalytic subunit of telomerase quiescent in healthy tissues) promoter element drives the gene expressions of *E1A* and *E1B*, proteins linked to the internal ribosome entry site (31). It has been shown that OBP-301 replicates effectively exclusively in human cancer cells (31).

The possible changes also include fibre viral capsid protein (32). The initial step of the adenoviral infection is the attachment of the virus to the Coxackie-adenovirus receptor (CAR). OBP- 405, a telomerase-specific replication-selective adenoviral agent, is a version of OBP-301 with a fibre modified to contain an RGD peptide that binds with high affinity to integrins on the cell surface, facilitating the CAR-independent virus entry (32). Delta-24-RGD adenovirus undergoes the 24 base pairs deletion in the E1A region that is responsible for binding the Rb protein. This deletion renders viral replication dependent on the inactivation of Rb and generates a tumour-selective, replication-competent virus that has been shown to induce an anticancer effect in some types of gliomas (33). Modification of viral hexon capsid protein into chimeric hexon with adenovirus serotype rare in nature has the potential to reduce hepatotoxicity, uptake in the liver and spleen, and innate immune response (34).

In recent years, several Oncolytic Adenoviruses (OAVs) armed with multiple regulatory elements combined, were developed. This further increased their specificity, efficacy, and ability to escape from patients’ immune systems, and made them the most remarkable among all OVs (15).

Currently, attempts are made to use OAVs as vectors carrying anticancer genes, the local expression that may result in tumour suppression, without affecting other cells, ultimately resulting in OAVs’ increased antitumour activity. One of the representatives of the newest generation of OAVs is OncoViron. Due to numerous modifications, its replication selectivity is regulated at both transcriptional and translational levels. The anticancer activity of viral structural proteins, the ability to infect cancer cells and avoid the neutralising antibodies, and the adsorption by hepatocytes are enhanced, and the killing effect on cancer cells is boosted by adding three types of anticancer immunomodulatory genes (15). OncoViron showed significant anticancer effects on its own and in combination with programmed death 1 (PD-1) antibody and chimeric antigen receptor (CAR) T cells on a variety of implanted solid tumour models, including breast cancer, in immunodeficient, immunocompetent, and humanized mice (15).

### 3.2 Protoparvovirus

H-1PV is a small single-stranded rat RNA virus that presents natural tropism to human cancer cells but does not replicate or induce cell lysis in non-transformed cells. No pre-existing immunity to H-1PV has been found in humans that acts as its advantage over OVs based on human pathogens (15). Various factors that are overexpressed in cancer cells are known to control H-1PV nuclear transfer. The research focussed on the identification of new cellular modulators has the potential to further favour the outcome of H-1PV treatment (15). Cancer cell lines derived from multiple tumours, including brain, pancreas, lung, cervical, colorectal, and breast cancers, as well as melanoma and osteosarcoma, are indeed susceptible to H-1PV infection and oncolysis (35). H-1 PV efficiency has also been shown in haematological diseases. H-1PV may cause apoptotic, non-apoptotic, and lysosome-dependent cell death. The latter is essential in glioma cells, resistant to conventional cytotoxic agents. Besides tumour lysis, the oncosuppressive effect of H-1PV results in the stimulation of both innate and adaptive immune responses (36). H-1PV has been tested in combination with conventional treatment, epigenetic modulators, apoptosis inducers, and angiogenic and immune-modulating drugs. The potential of some other rodent protoparvoviruses as anticancer therapeutics is also currently investigated in preclinical studies (37). Overall, the combination of enhanced viral entry, exploitation of dysregulated cellular pathways, defective antiviral responses, altered cell cycle regulation, and the tumour microenvironment contributes to the selective targeting and efficient oncolysis of cancer cells by Protoparvovirus H-1PV (38).

Protoparvovirus H-1PV targets cancer cells by exploiting overexpressed receptors like the transferrin and Heparan sulphate proteoglycan receptors, making them more susceptible to infection than non-cancerous cells (37).

Dysfunctional innate immune pathways and defective interferon responses in cancer cells create a favourable environment for viral replication and oncolysis (39).

By hijacking aberrant signalling pathways such as the Ras pathway, Protoparvovirus H-1PV facilitates its replication and spread within the tumour microenvironment, selectively killing cancer cells while sparing normal cells (40).

Factors like hypoxia, acidic pH, and immunosuppression in the tumour microenvironment enhance H-1PV replication in cancer cells while minimizing its impact on non-tumour cells (37).

Dysregulated cell cycle progression in cancer cells increases their susceptibility to viral infection and replication, with Protoparvovirus H-1PV preferring actively dividing cancer cells for infection and oncolysis (41).

### 3.3 Vaccinia virus

Vaccinia virus (VV) is a double-stranded DNA virus with a large genome that can be inserted with big fragments of transgenes and does not integrate into host cell chromosomes. Oncolytic VVs (OVVs) are engineered by knocking out the thymidine kinase (*TK*) gene and they can replicate exclusively in cancer cells (42). The focus of the research is to augment its oncolytic efficacy, which is naturally relatively low. Pexa-vec is an OVV that can activate the systemic immune response and inhibit tumour cells by expressing granulocyte-macrophage colony-stimulating factor (GM- CSF). It possesses features of two distinct infectious forms - intracellular mature virus (IMV) and extracellular enveloped virus (EEV). Such a characteristic allows its simultaneous intravenous and intratumoral injection, as well as evading neutralising antibodies (43). OVV has been recently used as a vector for personalised neoantigen immunotherapy against triple-negative breast cancer in a study assessing such a therapeutic approach (44).

### 3.4 Reovirus

Reovirus is a double-stranded RNA virus. Its replication is promoted in cells with an activated RAS pathway. The gain-of-function mutations, activating RAS signalling, are prevalent in cancers. Therefore, reovirus is a natural candidate for a therapeutic agent (45). Reovirus has oncolytic activity *in vitro* against multiple solid tumour types, including breast cancer (46). Reolysin is an unmodified wild- type oncolytic reovirus. In 2017, it received FDA approval for the treatment of metastatic breast cancer (46). Data from the Phase II trial for the treatment of advanced metastatic breast cancer showed that the combination of Reolysin and Paclitaxel significantly increased overall survival for about seven months (47). Clinical studies have demonstrated its effectiveness in combination with systemic anti-programmed cell death protein 1 (PD-1). In a murine breast cancer model, intratumoral reovirus increased PD-L1 expression on tumour cells, and combination reovirus/anti-PD-1 treatment improved survival by reducing Treg numbers and ameliorating tumour-specific cytotoxic T lymphocyte responses (48). Reovirus has also been used in combination with CD3-bispecific antibodies. Reovirus-induced IFN stimulated the recruitment of NK cells and reovirus-specific CD8+ T cells to the tumour site, while reovirus-specific effector T cells acted synergistically with CD3- bispecific antibodies, reducing the *in vivo* growth of several tumour types, including breast (46). Interestingly, this combination treatment was also effective against distant lesions that were not previously injected with reovirus. It suggests the possibility of using this therapy in case of metastatic disease (45). The advances in reovirus engineering have enabled the creation of oncolytic reoviruses that can antagonize inhibitory mechanisms within the TME. In particular, mutations in viral cell attachment protein *σ1* gene, have been incorporated to prevent proteolytic cleavage and inactivation of *σ1* by breast cancer-associated proteases (49).

### 3.5 Herpes simplex virus type I (HSV-1)

HSV is a DNA double-stranded neurotropic virus with a highly effective ability to infect. It is divided into two types - HSV-1 and HSV-2. The first one is commonly used for OV therapy. It has been widely used in cancer treatment. It is recognised as a potent activator of innate and adaptive immunity. Therapeutic forms of HSV-1 are obtained by modifying or deleting the genes that are crucial for viral replication in normal cells but not in tumour ones, such as thymidine kinase (*TK*), *ICP34.5* (required for viral replication in nerve cells), *ICP6* (coding the large subunit of HSV-1 ribonucleotide reductase), and *ICP47* (50–52). In addition, the knockout of *ICP47* prevents from inhibiting antigen presentation by MHC-1 and helps activate the host antitumour immune response (53). The examples of HSV-1 OVs are T-vec, with a knockout of *ICP34.5* and *ICP47*, and HSV1716, with deletion of double copies of *ICP34.5.* HSV1716 was approved for clinical trials in Europe in 1996 and has been used with satisfying results in the treatment of glioblastoma multiforme, melanoma, and head and neck squamous cell carcinoma (54). T-vec, also known as IMLYGIC or OncoVEX GM-CSF, proved to be effective and safe in a few Phase 1 clinical trials in patients with refractory breast cancer (55). It has been suggested that the retention of *ICP34.5* may be beneficial in some situations of IFN-dependent antiviral tumour status, as it can enhance the oncolytic effect and end the overall efficacy of OV. The alternative for direct deletion of the *ICP34.5* gene, proposed by researchers, is to control its expression by inserting into HSV a microRNA-responsive target element (56).

## 4 Discussion

### 4.1 Clinical use and observed adverse effects

The first OV was registered in 2004 in Latvia as a melanoma treatment. The OV is composed of the genetically unmodified Picarnoviridae family Enterovirus genus – Rigivir (57). Despite the standard procedure in treating melanoma with surgical resection, in metastatic melanoma, oncolytic viruses are showing promising effects. Melanoma has a heterogeneous presentation and can cause distant, dermal as well as visceral metastasis. The mechanism of OVs is appropriate for this type of cancer spread, as it causes the lysis of cancer cells and the lysis of infected malignant cells (58, 59). The retrospective studies from 2015 were performed in Latvia on 79 patients with stage IB, IIA, IIB, and IIC of melanoma after surgery (54). There was no statistically significant difference in the period of time for the patients to remain disease-free, however, the overall survival was prolonged among patients treated with Rigvir (54). In this study there were no significant side effects reported (54). Additionally, previous clinical trials reported side effects that were mild, completely reversible, and not causing an interruption in treatment, such as subfebrile temperature, pain in the location of the tumour, fatigue, sleepiness, and dyspepsia (55). Despite its registration in Latvia, Georgia, and Armenia, Rigvir was discontinued in mid-2019 due to manufacturing issues and then suspended for marketing authorization (60). Currently, there is a new product on the market - Imlygic registered by FDA in 2015 for advanced melanoma (7). Talimogene laherparepvec (T-VEC; Imlygic™), is a genetically modified herpes simplex virus, type 1. This is also the first OV to be approved by the FDA^2^ and EMA^3^. In the Phase III trial, 436 patients were enrolled, with unresected stage IIIB to IV melanoma but no metastases to the brain or bones. The durable response rate, overall survival, and objective response rate in patients treated with Imlygic were higher compared to the GM-CSF group (61). Adverse effects of using T-VEC were not severe, mostly presenting as flu-like mild symptoms: fatigue, chills, pyrexia, nausea, and local injection site reactions. Based on these significantly beneficial clinical results T-VEC was registered in monotherapy for curing advanced melanoma with unresectable cutaneous, subcutaneous, or nodal lesions after initial surgery by the FDA^2^ and the ATGA and for the treatment of stage III and IV M1a melanoma by the EMA^3^ (61–63).

In China in 2005 another OV, Oncorine, was approved by the National Medical Products Administration (NMPA) of China for treating nasopharyngeal carcinoma and advanced head and neck cancer (64). Oncorine is a genetically modified (deletion of *E1b55K*) human adenovirus type 5 called H101, which was developed by the Chinese company Sunway Biotech (65). In 2021 results of a new retrospective cohort study were published, with very promising clinical outcomes, using Oncorine as a treatment for advanced gastric carcinoma. 95 patients were divided into 3 groups (A=30, B=33, C= 32) and treated with Oncorine only (A), chemotherapy, and a combination of H101 and chemotherapy only (C). The results showed that control of lesions, progression-free survival, and overall survival were significantly higher in patients treated both with chemotherapy and Oncorine. What also was noted, is that side effects typical for chemotherapy such as nausea, vomiting, granulocytopenia, anemia, and hair loss occurred in patients from groups B and C (with no statistically significant difference between them) and more commonly than in group A. Pyrexia was observed mostly in patients from groups A and B. The study showed that Oncorine may be a therapeutic option for patients with gastric carcinoma in combination with chemotherapy (66). In 2022 the next retrospective study was carried out on the efficacy and safety of Oncorine in 29 patients with persistent, recurrent, or metastatic gynecologic malignancies: cervical cancer (22 cases), vaginal cancer (2 cases), vulvar cancer (3 cases) and ovarian cancer (2 cases). 22 patients responded to the treatment - significant tumour regression and reduction in necrotic tissue were observed as well as longer progression-free survival. Additionally, the objective response rate for radiotherapy was 72.4%, suggesting that Oncorine may increase the radiotherapy sensitivity (64). Side effects were similar to those that were previously reported, such as pyrexia, nausea, vomiting, fatigue, and pain with sometimes bleeding in the place of injection (60). There was one case reported of severe side effects - a rectovaginal fistula after Oncorine combined brachytherapy (60). Oncorine’s potential in the treatment of liver cancer, MPE, and pancreatic cancer is currently being investigated.

The newest registered OV is Delytac. This is a genetically engineered replication-competent herpes simplex virus type 1, called G47Δ or teserpaturev, approved in 2021 by the Japanese Ministry of Health, Labor and Welfare and developed by Daiichi Sankyo Co (67). Of 19 patients enrolled in this trial, 13 met the primary criterion of 1-year survival and the study was terminated earlier because of high efficacy achieved (68). 3 patients developed pyrexia but also severe side effects were reported such as death, cerebral infarction, hemiplegia, syncope, urinary tract infection, postprocedural infection, and subcutaneous abscess each in 1 patient (68).

Breast cancer is the most common cancer among women in Poland and the second cause of cancer-related deaths^4^. It has a relatively good prognosis if it is diagnosed and treated in early stages. On the other hand, when it is advanced and with metastases the treatment methods are limited and mortality rises due to complications of cancer itself as well as due to the treatment-related toxicity (69)^5^. The research in OV for breast cancer is now in the clinical trials phase and it showcases very promising effects as a future cancer treatment due to its ability to target only tumour cells (70) (71). In the preclinical and clinical trials, four virus groups (from seven groups of Baltimore classification) are mostly researched: group I (double-stranded DNA viruses), group III (double-stranded RNA viruses), group IV (single-stranded RNA viruses – positive-sense), and group V (single-stranded RNA viruses – negative-sense). Among these viruses there are naturally anti-neoplastic, those that are designed for tumour-selective replication, and those genetically modified to activate the immune system (71).

Currently, there is no OV registered for breast cancer treatment. **Table 3** represents OVs used in clinical trials (in monotherapy and combined therapy) tested on patients with breast cancer, however, there are a number of ongoing preclinical trials focusing on a variety of viruses from those four groups.

**Table 3:** Oncolytic viruses used in clinical trials (up to date, Dec 2023).

Baltimore classification	Virus group	Virus applied	Clinical outcome	Adverse reactions	References
**Group I** **Double-stranded DNA Viruses**	Adenovirus	Ad5/3-D24-GMCSF	3 out of 14 patients had tumour shrinkage or disease stabilization.	Most common: fever, fatigue, rigors, nausea, transient anemia, leukocytopenia. No serious AE is possibly related to the treatment.	(72)
		Ad5–Δ24–GMCSF	Disease stabilization in 3 of 7 patients with breast and colorectal cancer. One patient with advanced metastatic tumour, refractory to conventional therapies treated with a single round showed complete response in radiological evaluation.	Well tolerated, mostly flu-like symptoms: fever, chills, fatigue, and injection site pain.	(73, 74)
		RGD-4C (ICOVIR-7)	1 out of 3 patients presented stabilization of tumour markers, but at the endpoint nine of them showed effective response (mild, partial, non-complete).	No significant adverse effects occurred.	(75)
		ONYX-015+ etanercept	2 out of 2 patients with breast cancer showed progressive disease with a mean survival of only 125 days.	No significant AE: grade I and II fever within 24 h from administration	(76)
	Herpes simplex virus	Talimogene laherparepvec (T-VEC)	No partial or complete response was achieved but the disease stabilized in 1 patient (out of 14 with breast cancer).	The main side effects: grade 1 pyrexia with constitutional symptoms, 1 patient had grade 2 pyrexia with rigor, hypotension, and tachycardia. Other common: low-grade anorexia, nausea and vomiting, and fatigue. 2 patients developed abnormal liver function tests	(77)
		T-VEC+ neoadjuvant chemotherapy (NAC)	It was used on TNBC patients. In RCB-0 (complete pathologic response = pCR) and RCB-I (minimal residual disease) the 2-year disease-free was 89% with no recurrences. T-VEC plus NAC in TNBC may increase RCB0–1 rates.	Common AE: fevers, chills, headache, fatigue, and injection site pain. NAC as expected.	(78)
		HF10	In 6 patients with cutaneous or subcutaneous metastases from breast cancer 30% to 100% cancer cell death in histopathological evaluation.	No significant AE occurred	(79)
	Vaccinia virus	VVDD	2 out of 4 patients showed a partial antitumour response. It also showed remarkable vvDD selectivity for replication only in tumour cells.	No significant AE occurred (mostly fever, fatigue, nausea, vomiting); 1 severe adverse event - possibly related - pain in the rib 7 days after admission (no evidence of pulmonary problems).	
**Group III** **Double-stranded RNA Virus**	Reovirus	Pelareorep-wild form of reovirus	Applied on 2 breast cancer patients that resulted in partial oncolysis with 34% tumour shrinkage in one of them. Increased median overall survival (OS) in 74 advanced breast cancer patients.	Well-tolerated, grade 1-2 toxicities.	(80)
		Pelareorep and paclitaxel	74 women with previously treated metastatic breast cancer. The trial didn’t meet the primary endpoint which was progression-free survival, however, the combination resulted in a significant elongation of overall survival.	Well-tolerated.	(47)
**Group IV** **Positive sense** **Single-stranded RNA Virus**	No clinical studies on patients				
**Group V** **Negative-sense** **Single-stranded RNA Virus**	Newcastle Disease Viruses	PV701	1 out of 2 patients showed stable disease for more than 6 months.	No severe AE: the most common: flu-like symptoms, and injection site reactions.	(81)

Many preclinical trials focus on finding the OV against TNBC. TNBC is a highly aggressive cancer with a very poor response to treatment due to the lack of receptors for estrogen, progesterone, and human epidermal growth factor 2. Although the patients are currently treated with chemotherapy - the side effects are devastating, drug resistance occurs often, and the prognosis is poor. The difficulties in treatment and high heterogeneity in TNBC led to the beginning of many studies in order to find a more efficient and safer treatment (82).

Adenoviruses are the most studied OVs in breast cancer, so preclinical studies -with additional modification (antitumour and immune regulatory genes were inserted to enhance effects) - were performed also against TNBC (83). One of them is a recombinant type five adenovirus containing IL-24 gene (CNHK600-IL24). It significantly suppressed tumour growth in the nude mice model and improved survival in the metastatic model (84). Another OV which showed high efficiency against MDA-MB-435 cancer cells was G47Δ - oncolytic HSV (registered for Malignant Glioma). It presented high cytotoxicity against human breast cancer cells *in vitro* and in tumour xenografts *in vivo* (85). As for MDA-MB-231 TNBC cells VG9-IL-24 recombinant Vaccina virus presented promising effects. In the xenograft mouse model it showed efficiency in infecting and selectively killing breast cancer cells with no strong cytotoxicity to physiologic cells (86).

Recently (November 2023) a case study was published of a previously treated patient with mTNBC. The purpose was to evaluate the safety and efficiency of CHECKvacc - an oncolytic virus composed of CF33, a chimeric vaccinia poxvirus. The first intratumoral administration showed no immediate response, but later the patient underwent T-Dxd treatment and the tumour regressed significantly also disease-free survival was 10 months (87). This is just one of many examples where combined therapy shows the best effects. There is also an ongoing phase 1 clinical trial on Codalytic, this is the first codon-modified virus. In the preclinical trial, it was tested on a mouse model with implanted TNBC cells in monotherapy. After 3 weeks the tumour was reduced by 76% and the cure rate was 66% (88).

In clinical trials (listed in **Table 3**) the adverse effects in treating breast cancer and other types of cancer are mostly mild: flu-like symptoms such as low-grade fever, chills, nausea, and vomiting; with single severe adverse effects occurring.

Overall, OV therapy has been proven safe in many trials. Unfortunately, the inadequately effective outcomes of these trials and incomplete responses are not sufficient enough to use OVs as a monotherapy treatment for breast cancer. On the other hand, multi-approach strategies - combining OVs and chemotherapy, radiotherapy, or immunotherapy - provide better therapeutic effects and show great potential in future approaches to breast cancer treatment (69, 89, 90).

### 4.2 Immunological response to the oncolytic virus therapy

Cancer is not just a collection of rebellious cells. They need the support of a specific compartment of the tumour stroma called the TME. It provides many vital signals to support tumour growth and progression. We have long thought of the tumour stroma as a rather passive element of the bulky cancerous tumour, but it seems that its effects go far beyond blood supply and mechanical stabilization. It is now well established that the TME can influence almost every step of cancer growth. It plays a role in the initiation of cancer transformation, its growth, invasion, and ultimately metastasis. In rare cases, it can also induce spontaneous tumour regression.

The TME consists of cellular and non-cellular compartments. The tumour-associated stromal cell compartment contains immune cells as antigen-processing cells as macrophages (M1, M2), and antigen- presenting cells as dendritic cells (DC), and finally antigen-specific cells as T cells, both CD4 and CD8 subsets and B cells. In addition, other cells such as blood and lymphatic vessels with all their variety of cellular elements such as endothelial cells, cancer-associated fibroblasts, pericytes but also adjacent neuronal cells and adipocytes (91) play their role in the growth rate of cancer (92).

The non-cellular components of the TME are mainly matrix proteins, but also the often-neglected microbiome (93), which can be exploited by the tumour. Now that we know that the TME is involved in almost every step of cancer development and progression, it is not surprising that the development of therapies targeting the TME machinery is an emerging target for future cancer research. The cellular composition and dynamic function of the TME are not permanent but can vary greatly over time, depending on many local tissue factors reflecting the actual status of cancer growth. It depends on the tissue in which the cancer arises as well as the characteristics of the cancer cells, the stage of the tumour, and the clinical status of the patient.

TME usually reflects a normal immune response when the immune system is unable to eliminate an antigen. Under physiological conditions, the immune response initially attempts to eliminate the antigen. This situation occurs when a high burden of inflammatory cells can eliminate antigens at the site of immune action. It is associated with the presence of immunocompetent cells involved in antigen elimination, rich in CD4 Th1, T cytotoxic T, CD8, and NK cells. This situation is usually not present in TME without additional manipulations since the tumour grows when immune surveillance is impaired. If, over time, an antigen remains and the immune effort to eliminate it is useless, the immune response is redirected. It first changes to tolerate the antigen. The situation is equivalent to chronic inflammation, with an impaired ability to eliminate antigen but still control its spread. The cost is reduced immune surveillance. Finally, in the worst-case scenario, to protect the host, the immune system tries to physically isolate the source of the dangerous antigen, forming a physical barrier with extensive fibrosis. These last two situations are also indicative of an immunosuppressive microenvironment that could in the long term be the soil for cancer initiation and progression. The typical example is the so-called scar cancer in the lung, but many others can arise on the soil of chronic inflammation, e.g. liver cancer in hepatitis B infection, lung cancer following chronic irritations such as asbestosis and silicosis, and so on. These last two situations occur in cancers, therefore TME can finally be divided into distinct separate groups currently playing a significant role in clinical outcomes and response to therapy. The pro- and anti-tumourigenic effects of tumour-infiltrating immune competent cells can profoundly determine tumour progression and the success or failure of anti-cancer therapies.

Currently, based on the immunomorphological response to immunotherapy, TME can be classified into three main groups, referred to as: immune inflamed, immune excluded, and immune desert (94, 95). However, the response to immune checkpoint blockade immunotherapy has been linked to the degree of T cell infiltration (96, 97) in tumours with high tumour mutation burden (98) and neoantigen load (99), as well as tumour antigenicity (100, 101), which led to the elucidation of four distinct TME subgroups: immune-enriched fibrotic, immune-enriched non-fibrotic, fibrotic and immune-depleted (86–88).

Oncolytic virus treatment is an emerging and promising therapy that not only directly targets tumour cells but may also modify the TME towards its more immune-eliminating rather than immunosuppressive properties. Data confirms that these types of manipulations in an experimental model increased tumour vascular permeability, host leukocyte infiltration into tumours, and ultimately, tumour inflammation (102). A combination of OV and CAR-T cell therapy may stimulate naive T cells and enhance CAR-T efficacy in mice (103). A better understanding of the complex interactions between tumour cells and their stroma determines disease progression and is critical for the rational development of effective cancer therapy.

### 4.3 Side effects on non-tumour cells

The application of oncolytic viruses is not without potential side effects on non-tumour cells (104). While oncolytic viruses are designed to selectively target and destroy cancer cells, they may inadvertently impact neighbouring healthy cells (105). The mechanism of cytotoxic effects on non-tumour cells may be direct but can be mediated also by immunological responses (39). The activation of the immune system by viral infection can lead to the release of pro-inflammatory cytokines and chemokines, resulting in a local inflammatory response. While inflammation is a critical component of the antitumour immune response, excessive or prolonged inflammation can cause tissue damage and exacerbate pre-existing pathological conditions. Moreover, the activation of innate immune pathways, such as toll-like receptor signalling, may trigger immune-mediated cytotoxicity against non-infected cells, contributing to bystander effects (39). The risk is put also in off-target viral replication (106).

Moreover, oncolytic viruses may alter the tumour microenvironment, impacting the function and phenotype of stromal cells, endothelial cells, and immune cells, which can influence tumour progression and treatment outcomes (107).

Integrating strategies to mitigate off-target effects, such as engineering viruses with improved tumour selectivity or combining virotherapy with immunomodulatory agents, represents a promising approach to enhance the therapeutic index of oncolytic virus-based treatments (108, 109) The successes of no histological signs of viral induced toxicity for non-tumour bearing organs have been announced for the urokinase receptor (uPAR) retargeted oncolytic measles virus in syngeneic cancer models (110), Vstat120-expressing (RAMBO) oncolytic herpes simplex virus (oHSV) (111), novel combination oncolytic adenoviral gene therapy armed with Dm-dNK and CD40L for Breast Cancer (112), or cancer-specific targeting of a conditionally replicative adenovirus using mRNA translational control (105).

### 4.4 Potential biomarkers of response

The emergence of OV as a promising therapeutic approach in breast cancer has generated interest in identifying predictive biomarkers for treatment response (113). Biomarkers to predict response to OV in breast cancer patients are currently lacking but are essential for selecting patients who will most likely benefit from the treatment. Moreover, the rapidly expanding combination strategies force the finding of the biomarkers to match individual patients to their most promising treatment option (114).

One of the key determinants of response to OV in breast cancer treatment appears to be the immune composition of TME (115–117). Tumours exhibiting high infiltration of tumour- infiltrating lymphocytes (TILs) demonstrate a propensity for better responses to OV. TILs are indicative of pre-existing immunity which is essential for OV efficacy. Specifically, a robust presence of CD8+ T cells within the TME indicates a potentially favourable response (118, 119). Important to predict a response are not only higher levels of TILs but also the spatial distribution of TILs and their functional state (120). Moreover, the expression of immune checkpoint molecules like PD-L1 on tumour cells can indicate the likelihood of a favourable response when OVs are combined with immune checkpoint inhibitors (119).

Tumour Mutational Burden (TMB) serves as another potential biomarker influencing treatment outcomes. Tumours with increased mutational burdens harbour more neoantigens, rendering them more susceptible to immune recognition, thus potentially enhancing the response to oncolytic viruses (98, 121).

The infectivity and replication capacity of the oncolytic virus within tumour cells represent critical aspects for predicting treatment response. Techniques monitoring viral presence or replication within the tumour tissue might serve as valuable biomarkers (83, 122).

Genetic signatures linked to viral replication, immune response pathways, or susceptibility to viral infection might also contribute to predicting response to OVs in breast cancer. An example of this approach is the identification of realistic interferon (IFN)-mediated biomarkers to identify patients who most likely respond to virotherapy (123) as replication of OV is usually limited to cancer cells that have interferon (IFN) signalling defects. Upregulation of protein biomarkers such as IFN gamma may reflect immune induction and become an OV efficacy biomarker to improve the ability to select patients who do not exhibit resistance to virotherapy (124).

Analysing the cytokine profile within the TME provides insights into the immune response elicited by OVs. Elevated levels of specific cytokines may indicate a more favourable response to treatment. They may play a role in T-cell helper polarization in viral tolerability. The previous research described that the Th1 cytokine profile was expressed in pleural effusions of patients that responded to HSV1716 treatment for malignant pleural mesothelioma with low side effects, to be investigated as a biomarker for predictive response (125).

Serum markers such as carcinoembryonic antigen (CEA) have been also used to investigate the antitumour potential of a novel viral agent, an attenuated strain of measles virus deriving from the Edmonston vaccine lineage, genetically engineered to produce CEA against breast cancer. CEA production as the virus replicates can serve as a marker of viral gene expression (126). Therefore, CEA may serve as a low-risk method of detecting viral gene expression during treatment and could allow dose optimisation and individualization of treatment (126).

Advanced imaging techniques offer a non-invasive approach to monitor changes in the tumour microenvironment post-oncolytic virus treatment, potentially serving as a valuable tool for assessing treatment response. Examples of those may be organ-on-chip and tumour-on-chip microfluidic cell cultures (127).

Further research is imperative to establish the reliability and efficacy of these biomarkers in guiding the selection of patients likely to benefit from oncolytic virus therapy. The studies should be aimed at finding out how the ability of specific OVs to replicate in individual tumour cells is affected, and if and how it influences antitumour and antiviral action (89, 128).

### 4.5 Physical barriers

Several physical barriers can limit the delivery and effectiveness of the OVs and in our opinion, this topic requires a separate chapter in this review. Most of the therapies are delivered directly to the tumour. Such examples are adenovirus, poxvirus, HSV-1, measles, and reovirus which are delivered intramurally (129, 130). However, not all the tumours are available for direct delivery, because of their location. For example, the Seneca Valley Virus can be delivered to the bloodstream directly as it does not cause hemagglutination (131). Parvovirus H- 1PV can go through the blood-brain barrier and is applied in the treatment of glioblastoma multiforme (131). Another type of physical barrier is the extracellular matrix (ECM) in tumours is dense and contains impermeable for the viruses’ components, such as collagen and elastic fibres. For example, in pancreatic ductal adenocarcinomas (PDACs) ECM is so dense that can lead to interstitial hypertension (130) and can form a physical barrier for the delivery of the OVs (132). Other types of physical barriers associated with the TME are necrosis, calcification, hypoxia, acidosis, and increased proteolytic activity (130).

### 4.6 General risks of oncolytic virus therapy

#### 4.6.1 Immunogenicity

Immunogenicity is a critical consideration in the context of OV therapy, representing the capacity of the introduced viruses to stimulate an immune response within the host. Oncolytic viruses are designed to selectively replicate within cancer cells, triggering cell lysis and the release of tumour- associated antigens. This process is intended to provoke an immune response, engaging components such as T cells and natural killer (NK) cells. However, the effectiveness of this response depends on the ability of the immune system to recognise and mount a robust reaction against cancer cells. Successful oncolytic virus therapy relies on the activation of adaptive immune responses, particularly cytotoxic T lymphocytes (CTLs). These immune effectors play a central role in recognising and eliminating cancer cells. However, factors such as pre-existing immunity to the viral vector may influence the magnitude and efficacy of these responses (133).

#### 4.6.2 Off-target effects

Off-target effects refer to the unintended impact of OVs on non-cancerous or healthy cells within the body. Despite the selectivity engineered into these viruses, factors such as imperfect targeting mechanisms, interactions with the host immune system, or unexpected viral behaviour may lead to unintended consequences in off-target tissues. Off-target effects may manifest as localised toxicity in tissues surrounding the site of viral administration. This can include inflammation, tissue damage, or discomfort near the treatment site. In some cases, OVs may enter the bloodstream and disseminate throughout the body, potentially affecting distant organs and tissues. Systemic off-target effects can lead to more widespread adverse events. The activation of the immune system in response to viral infection may extend beyond the tumour site, leading to immune-mediated effects in healthy tissues. This could result in autoimmune-like reactions or inflammation in non-cancerous areas. This may be especially dangerous in immunocompetent patients (134).

#### 4.6.3 Inflammatory response

The introduction of OVs elicits an immune response aimed at recognising and eliminating foreign entities. While this response is essential for combating cancer, excessive or uncontrolled inflammatory reactions may lead to adverse effects. The inflammatory response can manifest both locally at the site of viral administration and systemically throughout the body. Locally, inflammation may cause redness, swelling, and pain at the injection site. Systemically, the release of inflammatory mediators into the bloodstream can lead to flu-like symptoms, fever, and malaise. The delicate balance between inducing an immune response against cancer cells and minimizing collateral damage to healthy tissues is pivotal for the therapy’s success. Excessive inflammation may not only compromise patient comfort but also impact the therapeutic effectiveness of oncolytic viruses by diverting the immune system’s attention away from cancer cells (135).

#### 4.6.4 Virus resistance

Virus resistance poses a significant challenge in oncolytic virus therapy, where cancer cells may develop mechanisms to evade viral infection and subsequent destruction. Cancer cells can develop resistance to oncolytic viruses through various mechanisms. These may include alterations in viral entry receptors, inhibition of viral replication, interference with the apoptotic pathways triggered by viral infection, and the evolution of antiviral immune responses within the TME (136).

### 4.7 Breast cancer-specific risks

Breast cancer exhibits substantial molecular and genetic heterogeneity, encompassing diverse subtypes such as luminal A/B, HER2-positive, and triple-negative breast cancer (TNBC). This heterogeneity influences disease progression, treatment response, and the overall clinical outcome. The diverse molecular landscape of breast cancer subtypes poses a challenge in designing oncolytic viruses with universal efficacy. Different subtypes may have distinct vulnerabilities and response patterns to viral infection, necessitating tailored approaches for each breast cancer subtype (90).

#### 4.7.1 Impact on healthy breast tissue

The proximity of healthy breast tissue to cancerous lesions raises concerns about potential off-target effects. Ensuring the selectivity of OVs for cancer cells while sparing normal tissue is critical in minimizing adverse effects and enhancing the safety profile of the therapy.

#### 4.7.2 Hormone receptor status

Hormone receptor status, including estrogen receptor (ER), progesterone receptor (PR), and human epidermal growth factor receptor 2 (HER2) expression, further complicates the landscape of oncolytic virus therapy. Subtypes with specific hormone receptor profiles may exhibit differential responses to viral infection, necessitating a nuanced approach to treatment planning (137).

#### 4.7.3 Combination therapies

OV therapy is often combined with other modalities such as chemotherapy, immunotherapy, or targeted therapies. Evaluating potential interactions and cumulative toxicities of these combined approaches is essential to mitigate risks and enhance therapeutic outcomes (55).

### 4.8 Limitations of the oncoviral therapy

OV therapy holds great potential in modern cancer therapy, however, the therapy with genetically modified viruses apart from its potential also shows limitations to overcome. One of the first obstacles is the delivery of the OVs. OVs can be administered intravenously, but it brings other barriers. OVs circulating in the bloodstream can be neutralised.

Major problems with systematic delivery are preexisting antibodies due to immunisation or previous oncolytic treatment. As Reovirus is commonly found in the environment, many people have antibodies against it which causes immunity to Reovirus and recombined OVs (138). In order to overcome the host’s immunity for different types of viruses, a lot of work still needs to be performed (139). Apart from antibodies circulating in the blood, there also are factors of the complement system, which after contact with the pathogen - activate and start the protease cascade, which leads to the deposition of the membrane attack complex (140).

There is also a risk of them not being guided directly into the tumour and nonspecific uptake by the lungs, liver or spleen, so only a small payload may be delivered to the tumour (139).

The next problem of intravenous administration is collapsed vasculature in the tumour, so the penetration can be insufficient, thus, the therapeutic dose will not be met (90). Currently, most of the OVs are injected directly into the tumour. The intratumoral administration can be difficult for the operator and painful for the patient, especially if the tumour is hardly accessible, as well as if the cancer cells are in several nodes dispersed in large areas - hence, not every metastasis might be equally reached (141). As for breast cancer treatment, the intratumoral administration of the OV is difficult only if the tumour is in a hardly accessed location or has already metastasized. The most optimal way of administration has not been defined yet, and also if OV should be used in monotherapy or in combination with other available forms of therapy.

Another difficulty in administration in solid tumours apart from delivering the OV to the patient itself, is the need to reach the tumour and spread in it, which can also cause limited efficacy against cancer cells (90). First, the OVs have to overcome the physical barriers (tissues) to get to the tumour. Secondly, intratumoral hypertension (caused by abnormal lymphatic networks, vascular hyperpermeability, and dense extracellular matrix) may obstruct viral infiltration, thus, the effectiveness of the OVs. (132, 136, 142).

The tumour is constructed with a great amount of extracellular matrix. Viruses, which are passively diffusing, may not fit through the strands. Limited penetration enables further tumour regions to grow regardless of administered OV (139). To improve tumour penetration various strategies are being developed such as pretreatment with enzymes or protein effectors (for example protease and relaxin) (143).

The next obstacle in OV therapy, which has been already recognised in the ongoing clinical trials, is the inadequate effectiveness in some types of tumours, when OVs are administered in monotherapy. In this case, combined therapy seems to be the best option, as confirmed by a growing number of studies with positive results (142). That has been reflected in a clinical trial on Oncorine, where the combination of Oncorine with chemotherapy resulted in better control of lesions, and elongated progression-free survival and overall survival in comparison to patients treated only with chemotherapy or only with Oncorine (121). Similarly, ONYX-015 with 5-fluorouracil used in clinical trials as a treatment for patients with recurrent head and neck cancer showed that after 6 months there was no progression in the tumours that responded to the OV treatment, while all of the tumours, treated only with chemotherapy, further progressing (144). ONYX-015 was also used in combination with etanercept for the treatment of patients with solid tumours. Patients with colon cancer achieved stable disease, while patients with breast cancer presented progression of the disease (76). The mechanism of OVs and drug combinations must be further understood and executed to find the best treatment solution. Currently, more research is required to present the most effective and safest treatment since most of the OV therapies are still in the early stages of development (136).

## 5 Future directions

Oncolytic virus therapy has emerged as a promising avenue in breast cancer treatment, showcasing remarkable potential in preclinical and early clinical trials. However, there are still challenges to overcome (**Table 4**).

**Table 4:** Challenges and future directions of developing OVs.

Challenges	Future Directions
Choosing the best treatment option for each particular patient based on tumour and patients’ characteristics	Indicating which OV will be the most suitable for patients by developing personalised viral platforms
Limited efficacy of OVs in monotherapy	Exploring combination therapies with chemotherapy or immunotherapy to enhance antitumour effect.
Delivering the OVs	Research focussed on improving delivery vectors to enhance tumour - specific targeting without loss of function and skipping barriers
Poor antitumour immune response	Developing interventions regulating checkpoints, modulating cytokine profiles, stimulating adaptive immune cells

The advent of precision medicine calls for tailoring OVs to individual patient profiles. Developing personalised viral platforms could involve genetic modifications or viral engineering to enhance tumour specificity, replication efficiency, and immune activation while mitigating adverse effects. Advancements in identifying predictive biomarkers for treatment response remain also urgent. Identifying biomarkers associated with the efficacy of OVs can aid in patient stratification, ensuring targeted therapies for individuals most likely to benefit.

An exciting frontier remains the synergy between OVs and conventional treatments like chemotherapy, radiotherapy, and immunotherapy. Exploring combination therapies can capitalize on their complementary mechanisms, potentially amplifying therapeutic efficacy and overcoming resistance.

It is also crucial to find strategies that restore the tumour microenvironment and bolster anti- tumour immune responses. This includes interventions that regulate immune checkpoints, modulate cytokine profiles, or stimulate adaptive immune cells within the tumour milieu.

Innovations in delivery systems to ensure efficient viral dissemination and penetration into tumour sites are also imperative. Engineering improved delivery vectors that enhance tumour-specific targeting while reducing off-target effects remains an active area of research.

A glycoprotein from others that has an affinity to the receptor can be inserted directly for enveloped viruses (145). An alternative approach is to use adapters that can bind both to the OV and the receptor (146). A promising approach are genetically engineered OV, with modification including deletions in the E1B region (147), E3B gene or for the PV deleting P1 coding region (replicons), A133G mutation in cis-acting replication element (CRE) (145). Off target effects are a concern especially for adenoviruses that have a high affinity to the liver (135). It has been reported that coagulation factor X (FX) binds to Ad5-hexon and enables transduction to the liver (148). Several modifications have been developed to circumferent the issue, such as constructing FX-binding ablated adenoviruses serotype 5 vectors (149). In the recent years. Recently the 3rd generation of OV has been introduced, e.g. OV with truncated CD19 (CD19t) protein for tumour-selective delivery (150).

As for many other research areas, a fast and efficient transition from preclinical success to clinical applicability remains the clue. Streamlining regulatory pathways and conducting robust clinical trials are essential steps towards obtaining approvals for OV therapies in breast cancer treatment.

The progress requires caring about the treatment’s long-term safety profiles. Establishing comprehensive monitoring mechanisms for potential adverse effects post-treatment is essential for ensuring patient safety and treatment optimisation.

The collaboration between researchers, clinicians, and pharmaceutical entities can facilitate resource-sharing, accelerate discoveries, and promote a collective effort towards advancing OV therapy. The future of oncolytic virus therapy in breast cancer treatment holds immense promise. Realizing these potential demands concerted interdisciplinary efforts, innovative strategies, and a commitment to translational research to revolutionize breast cancer management.

## Author contributions

HCh: Supervision, Writing – original draft, Writing – review & editing. AŚ: Writing – original draft, Writing – review & editing, Supervision. MM: Writing – original draft, Writing – review & editing. MBo: Writing – original draft, Writing – review & editing. MBr: Writing – original draft, Writing – review & editing. GD: Writing – original draft, Writing – review & editing. PD: Conceptualization, Data curation, Formal Analysis, Funding acquisition, Investigation, Methodology, Project administration, Resources, Software, Supervision, Validation, Visualization, Writing – original draft, Writing – review & editing. MW: Writing – original draft, Writing – review & editing.

## Footnotes

1 Food and Drug Administration. (2024). Alphabetical List of Licensed Establishments Including Product Approval Dates as of 01-JAN-2024. https://www.fda.gov/media/76356/download [Accessed January 16, 2024].

2 Food and Drug Administration. (2024). Alphabetical List of Licensed Establishments Including Product Approval Dates as of 01-JAN-2024. https://www.fda.gov/media/76356/download [Accessed January 16, 2024].

3 European Medicines Agency. (2022). Imlygic. https://www.ema.europa.eu/en/medicines/human/EPAR/imlygic [Accessed January 16, 2024]

4 Krajowy Rejest Nowotworów. Raporty. https://onkologia.org.pl/pl/raporty [Accessed January 16, 2024]

5 American Cancer Society. (2017). Breast Cancer. Facts & Figures 2017-2018. https://www.cancer.org/content/dam/cancer-org/research/cancer-facts-and-statistics/breast-cancer-facts-and-figures/breast-cancer-facts-and-figures-2017-2018.pdf [Access January 16, 2024]

## Glossary

**Table:**

5-FC	5-fluorocytosine
5-FU	5-fluorouracil
5-FUMP	5-fluorouridine monophosphate
AdV	Adenovirus
APCs	Antigen Presenting Cells
CCL	chemokine (C-C motif) ligand
CD	cytosine Deaminase
CEA	carcinoembryonic antigen
CTL	cytotoxic T lymphocyte
CXCL	chemokine (C-X-C motif) ligand
ECM	extracellular matrix
EEV	extracellular enveloped virus
ER	estrogen receptor
GM-CSF	granulocyte-macrophage colony-stimulating factor
HER2	human epidermal growth factor receptor 2
HSV-1	herpes simplex virus type I
HSV-10	herpes simplex virus type 10
HSV-2	herpes simplex virus type 2
IFN	interferon
IL	interleukin
IMV	intracellular mature virus
MMP-9	matrix metallopeptidase 9
MV	Measles virus
NK	natural killer
NMPA	National Medical Products Administration
OAV	oncolytic adenoviruses
OS	overall survival
OV	oncolytic virus
OVV	oncolytic vaccinia virus
PD-1	programmed cell death protein 1
PDAC	pancreatic ductal adenocarcinoma
PH20	hyaluronidase 5
PR	progesterone receptor
PSA	prostate-specific antigen
PTEN	phosphatase and tensin homolog
SMAC	second mitochondria-derived activator of caspases
T-VEC	Talimogene laherparepvec
TAAs	Tumour-Associated Antigens
TIL	tumour-infiltrating lymphocyte
TK	thymidine kinase
TMB	Tumour Mutational Burden
TME	tumour microenvironment
TNBC	triple-negative breast cancer
TRAIL	TNF-related apoptosis-inducing ligand
VEGF	vascular endothelial growth factor
VV	vaccinia virus
